# Economic evaluation alongside pragmatic randomised trials: developing a standard operating procedure for clinical trials units

**DOI:** 10.1186/1745-6215-9-64

**Published:** 2008-11-17

**Authors:** Rhiannon T Edwards, Barry Hounsome, Pat Linck, Ian T Russell

**Affiliations:** 1 Centre for Economics and Policy in Health, Institute of Medical and Social Care Research, Bangor University, Dean Street, Bangor, UK; 2 North Wales Organisation for Randomised Trials in Health and Social Care, Institute of Medical and Social Care Research, Bangor University, Ardudwy, Normal Site, Holyhead Road, Bangor, UK

## Abstract

**Background:**

There is wide recognition that pragmatic randomised trials are the best vehicle for economic evaluation. This is because trials provide the best chance of ensuring internal validity, not least through the rigorous prospective collection of patient-specific data. Furthermore the marginal cost of collecting economic data alongside clinical data is typically modest. UK Clinical Research Collaboration (UKCRC) does not require a standard operating procedure (SOP) for economic evaluation as a prerequisite for trial unit registration. We judge that such a SOP facilitates the integration of health economics into trials.

**Methods:**

A collaboration between health economists and trialists at Bangor University led to the development of a SOP for economic evaluation alongside pragmatic trials, in addition to the twenty SOPs required by UKCRC for registration, which include randomisation, data management and statistical analysis.

**Results:**

Our recent telephone survey suggests that no other UKCRC-registered trials unit currently has an economic SOP.

**Conclusion:**

We argue that UKCRC should require, from all Trials Units undertaking economic evaluation and seeking registration or re-registration, a SOP for economic evaluation as one of their portfolio of supporting SOPs.

## Background

There is wide recognition that pragmatic randomised trials are the best vehicle for economic evaluation [1-4]. This is because trials provide the best chance of ensuring internal validity, not least through the rigorous prospective collection of patient-specific data. Furthermore the marginal cost of collecting economic data alongside clinical data is typically modest [2].

Several text books and journal articles define best practice for economic evaluation alongside clinical trials. However, meeting the defined standards and integrating health economics into trial protocols and procedures is still a challenge. For example Drummond et al add a survival guide for health economists to the latest edition of their text book [2]!

## Methods

The health economics team at Bangor University collaborated with the North Wales Organisation for Randomised Trials in Health [NWORTH], the regional trials unit, in its application to the UK Clinical Research Collaboration [UKCRC] in 2007 for full registration, offering health economics as an additional methodological capability. This collaboration led to the development of a standard operating procedure [SOP] for economic evaluation alongside pragmatic trials, in addition to the twenty SOPs required by UKCRC for registration, which include randomisation, data management and statistical analysis. Though UKCRC does not require a SOP for economic evaluation as a prerequisite for trial unit registration, we judge that such a SOP facilitates the integration of health economics into trials. Our recent telephone survey suggests that no other UKCRC-registered trials unit currently has a similar SOP available.

We had previously undertaken economic evaluations alongside pragmatic RCTs [5,6]. So we based this SOP on previous experience and current health economics resources. Our SOP needed to cover the range of trials to which we currently contribute economic evaluation. These include trials in clinical medicine, for example COGNATE (trial of endoscopic ultrasonography in gastro-oesophageal cancer – ISRCTN 01444215) and FolATED (trial of folate as adjunct to anti-depressant therapy ISRCTN 37558856) [7]; trials of psycho-social interventions, notably RemCare (trial of reminiscence therapy for patients with dementia – ISRCTN 42430123); and public health trials, notably CHARISMA (trial of housing improvements for children with asthma – ISRCTN 13912429). The current trials all evaluate 'complex interventions' in many centres by adopting a pragmatic whole-system approach to trial design and conduct, and hence to the concomitant design and conduct of economic evaluation [8].

Economists and trialists work closely together to put health economics at the core of trials co-ordinated from Bangor. For example we appointed a health economist as trial manager and as health economist to the RemCare trial. This has enabled us to integrate economic evaluation into trial protocol preparation, design of information sheet and consent form, application for ethical approval, and early preparation of analysis plans and of publication policy. In this way we are meeting the defined standards for the design, conduct and reporting of economic evaluations [1-4].

**Figure 1:**
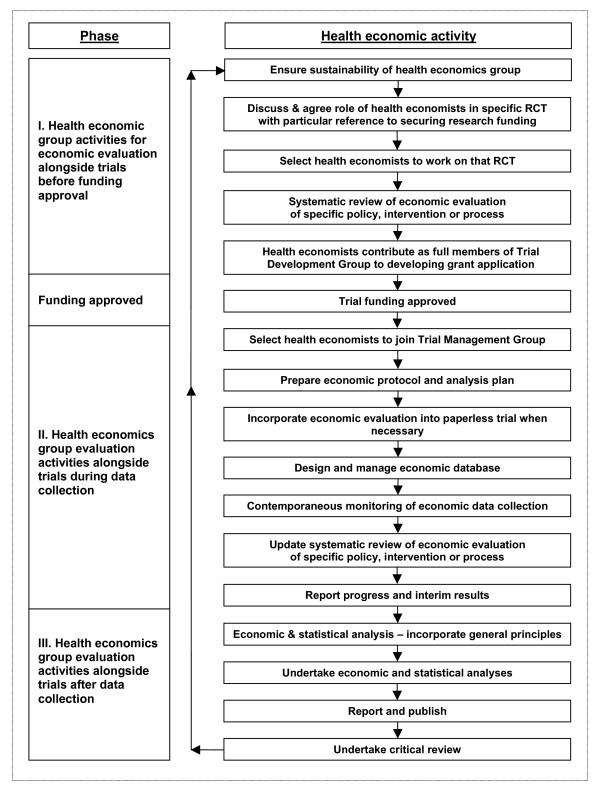
Flowchart illustrating a standard operating procedure for economic evaluation alongside trials.

The Bangor SOP [see Additional file 1] for economic evaluation alongside pragmatic trials, summarised in Figure 1, divides economic evaluation activities into three phases: those preceding funding, thus requiring the development and sustainability of a health economics research group, notably pre-funding protocol development; data collection and management; and economic and statistical analysis leading to publication. Phase I ensures that health economics is integral to the development of rigorous and comprehensive protocols. Phase II seeks to achieve a comprehensive data set in which clinical and economic data complement each other. Phase III completes the symbiotic process of clinical and economic evaluation. This SOP is being considered for adoption by other trials units in Wales. The SOP is also available electronically  and 

## Discussion

SOPs cannot themselves overcome the two main limitations of conducting economic evaluations alongside clinical trials – namely the length of follow-up in a typical trial and the limited number of treatment options compared [2-4]. Nevertheless, Drummond et al have argued that standard reporting frameworks for economic evaluation promote transparency, improve comparability of published studies and stimulate economists to address challenging methodological issues [2,9]. Similarly, we have found that the development of a SOP for economic evaluations alongside pragmatic trials managed by a registered trials unit improve trial conduct and hence the validity and generalisability of results. Above all, this process leads to a research culture in which clinical and economic findings receive equal credit.

## Conclusion

Thus, we argue that UKCRC should require from all Trials Units undertaking economic evaluation, and seeking registration or re-registration, a SOP for economic evaluation as one of their portfolio of supporting SOPs.

## Competing interests

The authors declare that they have no competing interests.

## Authors' contributions

RTE and BH conceived the drafted the standard operating procedure and manuscript. PL and ITR critically reviewed and revised the standard operating procedure and manuscript. All authors read and approved the final manuscript.

## Supplementary Material

Additional file 1 **CEPhI-NWORTH economic evaluation SOP.** Economic evaluation standard operating procedure. Click here for file
