# Supplementary material for: Economic evaluation alongside pragmatic randomised trials: developing a standard operating procedure for clinical trials units
Source: Trials. 2008 Nov 17;9:64. doi: 10.1186/1745-6215-9-64 (PMC2615739; doi:10.1186/1745-6215-9-64)
Supplement: Additional file 1 — CEPhI-NWORTH economic evaluation SOP. Economic evaluation standard operating procedure. [file 1745-6215-9-64-S1.doc]

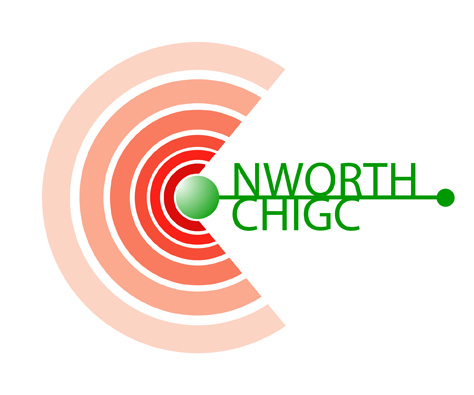

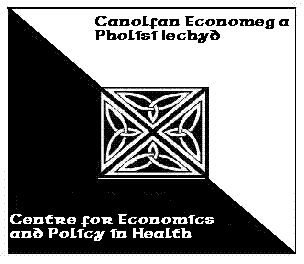


CEPhI Economic Evaluation Standard Operating Procedure (General EE.1) for NWORTH adopted trials and other trials randomised by NWORTH.

**SOP number**: EE.1.1

SOP name: NWORTH07/21healtheconomics Version 2

Authorship Team: Rhiannon Tudor Edwards, Barry Hounsome, Pat Linck.

Review Date_____________________

**Designated NWORTH member (DNM) approval**:

Name: Ian Russell

Signature___________________________Date________________________

**1^st^ Author approval**

Name: Rhiannon Tudor Edwards

Signature____________________________Date_______________________

**NWORTH trial unit manager approval:**  Name: Rhiannon Whitaker

Signature __________________________ Date of Approval______________

**Authorised for implementation by: the Director of NWORTH**

Signature ___________________________ Date of Approval_____________

Effective Date__________________________________________________

| **Revision record** | | |
| --- | --- | --- |
| **Version Number** | **Effective Date** | **Reason for Change** |
|  |  |  |
|  |  |  |
|  |  |  |

# Section 1: Table of Contents

[Section 1: Table of Contents 2](#__RefHeading___Toc213144881)

[Section 2: Glossary of Terms 3](#__RefHeading___Toc213144882)

[Section 3: Introduction 5](#__RefHeading___Toc213144883)

[Section 4: Purpose 6](#__RefHeading___Toc213144884)

[Section 5: Users 7](#__RefHeading___Toc213144885)

[Section 6: Procedures 8](#__RefHeading___Toc213144886)

[6.1 Overview 8](#__RefHeading___Toc213144887)

[*6.1.1* *CEPhI Sustainability* 8](#__RefHeading___Toc213144888)

[*6.1.2* *Role of CEPhI in securing research funding for trials* 8](#__RefHeading___Toc213144889)

[6.2 CEPhI activities for economic evaluation alongside a RCT prior to funding approval 9](#__RefHeading___Toc213144890)

[*6.2.1* *Select CEPhI staff to be involved in the economic evaluation alongside a specific RCT* 9](#__RefHeading___Toc213144891)

[*6.2.2* *Review health economics literature (SOP EE.6)* 9](#__RefHeading___Toc213144892)

[*6.2.3* *Contribute to Trial Development Group* 9](#__RefHeading___Toc213144893)

[6.3 CEPhI economic evaluation activities alongside a RCT during the data collection period in a trial 10](#__RefHeading___Toc213144894)

[6.3.1 Select health economist(s) to join the Trial Management Group 10](#__RefHeading___Toc213144895)

[*6.3.2* *Prepare an Economic Evaluation Analysis Plan (SOP EE.3)* 10](#__RefHeading___Toc213144896)

[*6.3.3* *Incorporate economic evaluation into paperless trials when necessary (SOP EE.4)* 11](#__RefHeading___Toc213144897)

[*6.3.4* *Monitor collection of health economics data* 11](#__RefHeading___Toc213144898)

[*6.3.5* *Design and manage economic database (SOP EE.7)* 11](#__RefHeading___Toc213144899)

[*6.3.6* *Conduct a systematic review of the literature if not conducted under (6.2.2); otherwise update the systematic review (SOP EE.6)* 11](#__RefHeading___Toc213144900)

[*6.3.7* *Presentation of progress and interim results to trial team and funding body.* 11](#__RefHeading___Toc213144901)

[6.4 CEPhI activities for economic evaluation alongside a RCT after the data collection period in a trial. 11](#__RefHeading___Toc213144902)

[*6.4.1* *Economic/statistical analysis – incorporate general principles* 11](#__RefHeading___Toc213144903)

[*6.4.2* *Economic/statistical analysis – undertake analyses* 12](#__RefHeading___Toc213144904)

[*6.4.3* *Report and publish* 13](#__RefHeading___Toc213144905)

[*6.4.4* *Critical review* 13](#__RefHeading___Toc213144906)

[Figure 1: Flow diagram for health economic evaluation in RCTs 14](#__RefHeading___Toc213144907)

[Section 7: Staff Training and Development 15](#__RefHeading___Toc213144908)

[Section 8: References 16](#__RefHeading___Toc213144909)

[Section 9: Referenced SOPs 17](#__RefHeading___Toc213144910)

[Section 10: Appendices 18](#__RefHeading___Toc213144911)

[Appendix 1: Training for this SOP 18](#__RefHeading___Toc213144912)

# Section 2: Glossary of Terms

CEA Cost Effectiveness Analysis - a form of economic analysis in which the results are expressed as a ratio of cost per unit of health outcome, the latter normally being expressed in ‘natural units’ (e.g. mm Hg change in blood pressure, symptom free days). (OHE, 2008).

CEAC Cost Effectiveness Acceptability Curve.

CCA Cost Consequences Analysis - a form of economic analysis in which the outcomes (of which a variety of measures are normally presented) are reported separately from costs (OHE, 2008).

CEPhI Canolfan Economeg a Pholisi Iechyd/Centre for Economics and Policy in Health.

CSRI Client Service Receipt Inventory (Knapp et al 2006).

CTU Clinical Trial Unit - The organisation running the trial. While this will often be NWORTH, it may also be a trials unit from another region.

CUA Cost Utility Analysis - a form of economic analysis in which the results are expressed in terms of cost per QALY gained (OHE, 2008).

Economic Guarantor The person who guarantees, and takes responsibility for, the economic rigour of the trial, its analysis, and its publication.

EE Economic Evaluation - a comparative analysis of two or more alternatives in terms of their costs and benefits (OHE, 2008).

ICER Incremental Cost Effectiveness Ratio – the difference in costs between one intervention and an alternative, divided by the difference in outcomes (OHE, 2008).

Methodological Guarantor The person who guarantees, and takes responsibility for, the methodological rigor of the trial, its analysis, and its publication.

NICE National Institute for Health and Clinical Excellence.

NMB Net Monetary Benefit.

NWORTH North Wales Organisation for Randomised Trials in Health (and Social Care).

QALY Quality Adjusted Life Year - a measure of the benefit of health care combining the impact on both expected length of life and quality of life (OHE, 2008).

SOP Standard Operating Procedure.

Statistical Guarantor The person who guarantees, and takes responsibility for, the statistical rigour of the trial, its analysis, and its publication.

WHESS Welsh Health Economics Support Service.

# Section 3: Introduction

This document forms part of a sub-set of standard operating procedures for NWORTH. This sub-set covers the conduct of economic evaluation alongside randomised controlled trials in health and social care (NWORTH/CEPhI.SOP.EE.1 - 9 ). These have been developed by staff at the Centre for Economics and Policy in Health. The Centre provides rigorous health economics support, including economic evaluation alongside clinical trials, to NWORTH. This general overarching SOP (SOP.EE.1) identifies the roles, responsibilities and actions of the personnel involved in all aspects of economic evaluation alongside clinical trials, be these managed by or approved by NWORTH.

This SOP is guided by key references in the field of health economics specifically NICE Technical Guidance 2008 and other relevant guidance (Drummond and McGuire, 2001; Donaldson *et al*., 2002; Eggar *et al*., 2003; Drummond *et al*., 2005; Ramsey, *et al*., 2005; Glick *et al*., 2007; NICE, 2007).

Section 4: Purpose

The purpose of this general, overarching SOP is to outline stages in the conduct of economic evaluation alongside clinical trials, and to set other specific SOPs within an overall methodological context. These other SOPs are listed in Section 9, page 16.

# Section 5: Users

The personnel responsible for the delivery of robust economic evaluation alongside clinical trials through NWORTH/CEPhI are as follows:

**Chief Investigator**

The Chief Investigator will work with the Director/Deputy Director of CEPhI at all stages of the trial. These two will take joint responsibility for complying with the requirements of the trial protocol and this SOP.

**Senior Trial Economist and Guarantor**

The Director or Deputy Director of CEPhI will act as guarantor through undertaking the design, monitoring of conduct, quality, final analysis, and manuscript preparation for publication of economic evaluation alongside clinical trials. In general a senior pharmacoeconomist will act as guarantor for trials of a pharmaceutical nature, while a senior health economist will act as guarantor for non pharmaceutical trials, e.g. cancer trials, public health, psycho-social, and other health/social care trials. The post holder will liaise with the Trial Statistician throughout all stages of the trial.

**Trial Health Economist**

Trial Economists will be responsible for attending NWORTH meetings when necessary, but will be in regular attendance at specific trial team meetings. They will be responsible for the day to day running of the economic evaluation alongside a specific clinical trial, liaison with the NWORTH team, liaison with research staff collecting data at multiple sites, data entry, data cleaning and presentation of updates at meetings. They will be fully supported by the Senior Trial Economist.

Whenever a Trial Economist is also the trial co-ordinator, the post holder will be responsible for managing one large multi-centre trial and undertaking health economics for that trial in addition to providing specialised support to other trials (e.g. modelling), time permitting. The post holder will be responsible for attending all NWORTH meetings, reporting to the Director of CEPhI on liaison between NWORTH and CEPhI. The post holder will liaise with the Trial Statistician throughout all stages of the trial.

**Trial Statistician**

The Trial Statistician will liaise with the Senior Trial Economist to ensure that the statistical and economic plans of analyses are fully integrated at all stages of the trial, including publication.

**Administrative support**

The CEPhI administrator will undertake routine administrative tasks to support the monitoring of economic evaluation alongside clinical trials, arranging meetings, filing documents and helping with preparation of articles for peer reviewed publication. Where administrative support resources have been built into the costing of a trial he/she will undertake administrative tasks for specific trials.

# Section 6: Procedures

When reading this section, please refer to the flow diagram (Appendix 1, Page 17) that summarises the processes involved in integrating health economics into a clinical trial.

## 6.1 Overview

### *6.1.1 CEPhI Sustainability*

Research staff recruitment, training, and retention will aim to ensure that CEPhI has a sustainable and skilled team with the necessary health economics expertise to deliver rigorous and consistent, state of the art, economic evaluation alongside RCTs. CEPhI has a policy of prioritising the extension of current staff contracts over the appointment of new staff in order to promote career planning, employment security and a critical mass of increasingly experienced staff. This means that research staff should be flexible and willing to move from one project to another. CEPhI is also prepared to support researchers working within IMSCaR, with good analytical skills (e.g. SPSS and Excel) wishing to move into the field of economic evaluation. In addition, CEPhI will pursue sustainability through:

- Seeking national (UK) accreditation for economic evaluation alongside RCTs.
- Enhancing its national and international profile (e.g. promotional activities, networking, website, publications and presentations at relevant conferences).
- Seeking resources to enable research staff to undertake short courses (e.g. in economic evaluation alongside RCTs, economic modelling, and good clinical practice).

### *6.1.2 Role of CEPhI in securing research funding for trials*

Inquiries received by CEPhI and originating in Wales, for health economics support alongside proposed RCTs, will be logged in Bangor through the Welsh Health Economics Support Service (WHESS). All enquiries will be considered promptly by the Director and/or Deputy Director of CEPhI. Where a proposal is considered to be worthy of further development the Director or Deputy Director, and other staff, as appropriate, will work with the Trial Development Group to develop/write the grant application and ensure the adequate resourcing of the economic evaluation (SOP EE.2) alongside a specific clinical trial. More specifically:

- The Director or Deputy Director of CEPhI will be co-applicants on trial grant applications, acting as Senior Trial Economist and guarantor for the economic analysis.
- Where possible, CEPhI research staff with relevant experience and expertise, who have helped build up a grant proposal and who are expected to act as named researcher, will also be offered co-applicant status. This will not always be possible, but their contribution will be recognised in publications.
- The Director or Deputy Director of CEPhI will need an opportunity to specify the health economics resources required for any trial protocol prior to grant application submission.

## 6.2 CEPhI activities for economic evaluation alongside a RCT prior to funding approval

### *6.2.1 Select CEPhI staff to be involved in the economic evaluation alongside a specific RCT*

Two staff selections need to be made:

- Senior Trial Economist/Guarantor. The Director or Deputy Director of CEPhI will act as Senior Trial Economist/Guarantor for the design, monitoring of conduct, quality and final analysis and preparation of manuscripts for publication of economic evaluation alongside clinical trials. In general the lead pharmacoeconomist will act as guarantor for trials of a pharmaceutical nature, while a senior health economist will act as guarantor for non-pharmaceutical trials (e.g. cancer trials, public health, psycho-social, and other health/social care trials).
- Trial health economist. Where possible, in CEPhI, an attempt will be made to match staff interests, experience, contract funding and workload, against trials. Where such a match does not appear to be working effectively, the Director and Deputy Director will negotiate, through consultation with the researcher concerned and the CTU (e.g. NWORTH) director and manager, staff changes in the interests of effective completion of the trial.

### *6.2.2 Review health economics literature (SOP EE.6)*

A comprehensive review (or, systematic review if time and resources allow) of the health economics literature will be conducted for all trials according to established guidelines (Donaldson *et al*., 2002; Eggar *et al*., 2003). The literature review will be updated during the course of the trial.

### *6.2.3 Contribute to Trial Development Group*

A number of important choices regarding the economic evaluation will have to be made at the grant writing/trial protocol development stage of a bid. These include:

- Defining the economic question of interest in a trial. The economic question will be drafted and specified in the economic plan of analysis and based on discussions with the CTU (e.g. NWORTH) and the wider trial team.
- Choice of a method(s) of economic evaluation appropriate to the trial. The choice of method of economic analysis (CEA, CUA, CCA) will be guided by: Drummond and McGuire, 2001; Donaldson *et al*., 2002; Eggar *et al*., 2003; Drummond *et al*., 2005; Ramsey, *et al*., 2005; Glick *et al*., 2007; NICE, 2007.
- Choice of measure of outcome/effect/consequence. In consultation with the CTU (e.g. NWORTH) and the wider trial team, the choice of measure of outcome will be guided by: Drummond and McGuire, 2001; Donaldson *et al*., 2002; Eggar *et al*., 2003; Drummond *et al*., 2005; Ramsey, *et al*., 2005; Glick *et al*., 2007; NICE, 2007.
- Choice of perspective of analysis. We will adopt a multi agency public sector or NHS/PSS perspective where possible. Where this is not relevant we will adopt an NHS perspective (NICE, 2007).
- Selection of type and range of costs to be measured. The choice of type and range of costs will be guided by the perspective of the analysis and: Drummond and McGuire, 2001; Donaldson *et al*., 2002; Eggar *et al*., 2003; Drummond *et al*., 2005; Ramsey, *et al*., 2005; Glick *et al*., 2007; NICE, 2007.
- Choice of method of measurement of health/social care utilisation (i.e. patient records versus patient recall via CSRI versus prospective data capture via CRF). Explicit consent for economic evaluation will be incorporated into consent forms for study participants. Where possible, information on the frequency and type of health and social care contacts will be obtained through asking trial participants to recall service contacts over no more than the last 6 months. This will be achieved by use of CSRI (Knapp *et al*., 2006) and/or a simple diary. In paperless trials, secondary care service contacts will be recorded directly by research nurses at trial centres and incorporated into the NWORTH trial database. Where resources allow, triangulation or verification of patient recall of service use will be conducted in a sub-sample of study participants through a patient record review in primary and secondary care settings.
- Choice of source of costs. Services will be valued using national costs (£ Sterling for the UK, in the most recently available year). The possibility of using routine data sources will be explored at the outset.
- Determining the method of collecting prescribing data (SOP EE.5). A collective decision, guided by the pharmacoeconomist trial guarantor, will negotiate whether some or all of prescribing information is collected directly from hospital notes and/or primary care in a trial. Attention will focus on drugs that are relevant to the disease under investigation, and to high cost or high volume prescribed drugs. Though CSRIs provide a robust method for collecting information on service contacts in the short term, patient recall of prescribed drugs is unreliable.

## 6.3 CEPhI economic evaluation activities alongside a RCT during the data collection period in a trial

## 6.3.1 Select health economist(s) to join the Trial Management Group

The Senior Trial Economist/Guarantor and Trial Economist will be members of the Trial Management Group and at least one of them will regularly attend meetings.

### *6.3.2 Prepare an Economic Evaluation Analysis Plan (SOP EE.3)*

This may be written as part of a grant application or to complement a grant application or trial protocol. An economic analysis plan will be produced for each trial based on: Drummond and McGuire, 2001; Donaldson *et al*., 2002; Eggar *et al*., 2003; Drummond *et al*., 2005; Ramsey, *et al*., 2005; Glick *et al*., 2007; NICE, 2007. This plan will be written and then, following consultation, signed off by The Director and/or Deputy Director of CEPhI and the Director of NWORTH, prior to or during the first 3 months of a trial.

### *6.3.3 Incorporate economic evaluation into paperless trials when necessary (SOP EE.4)*

CEPhI staff will help with the design of the data collection programme template at the beginning of the trial, so that economic data collection is fully integrated with clinical data collection. In paperless trials, secondary care service contacts will be recorded directly by research nurses at trial centres. CEPhI research staff will liaise with research nurses at multiple sites, throughout the data collection period, to ensure as complete set of service utilisation data as possible.

### *6.3.4 Monitor collection of health economics data*

The trial health economist will liaise regularly with the CTU (e.g. NWORTH) trial team throughout the data collection period. Principal contacts will be with the trial coordinator and data manager. The Senior Trial Economist/Guarantor should be a member of the trial management group.

### *6.3.5 Design and manage economic database (SOP EE.7)*

CEPhI, in collaboration with the Trial Statistician, will manage the economic data in an appropriate software package in accordance with CTU (e.g. NWORTH) SOPs and the Data Protection act 1998. CEPhI will take responsibility for putting data in an appropriate format in collaboration with the Trial Statistician.

### *6.3.6 Conduct a systematic review of the literature if not conducted under (6.2.2); otherwise update the systematic review (SOP EE.6)*

A systematic review of the literature will be conducted, if not already undertaken earlier (under 6.2.2). If a systematic review has already been conducted this should be updated (on an on-going basis, at least annually).

### *6.3.7 Presentation of progress and interim results to trial team and funding body.*

The Trial Economist and/or Senior Trial Economist/Guarantor will be available to attend key progress meetings with funding bodies and regular trial management meetings.

## 6.4 CEPhI activities for economic evaluation alongside a RCT after the data collection period in a trial.

### *6.4.1 Economic/statistical analysis – incorporate general principles*

Common to each method (CEA, CUA, CCA), the economic/statistical analysis will:

- Use an intention to treat approach.
- Address consistently missing or censored data, in conjunction with the NWORTH statistician. The procedure for dealing with missing data will be as defined in the NWORTH Statistical SOP (NWORTH07/15statistics).

The procedure for dealing with censored data will take account of Bang & Tsiatis (2000).”

- Fully address issues of uncertainty by applying the standard methods of uncertainty analysis (e.g. bootstrapping for calculation of CEAC and confidence intervals) (Glick *et al*. 2007; Groot Koerkamp *et al*., 2007; NICE, 2007).
- Use an appropriate time horizon.
- Apply an appropriate real discount rate to costs and benefits.
- Apply an appropriate ceiling ratio in CEA and CUA. This will be the £20,000 and £30,000 thresholds used by NICE in cost per QALY calculations, but will need an objective, pragmatic justification in disease specific measures used in CEA.

### *6.4.2 Economic/statistical analysis – undertake analyses*

In principle the following analyses will be undertaken:

- Costs for each trial participant will be calculated (SOP EE.8). Frequency of service use will be multiplied by the unit cost to produce a total cost per patient.
- In preparation for reports and publication, routine frequencies and costs will be reported to show a clear, traceable route to final cost figures used in CEA analysis. This will include in tabular form, mean and SD costs for intervention and control groups by health sector e.g. primary, secondary, special intervention and drugs.
- A plausibility check on the dataset and preliminary findings will be undertaken in collaboration with NWORTH (SOP EE.7 and EE.8). Prior to ICER/statistical analysis, at the end of the trial, and at regular intervals during the trial, the data set will be reviewed. There will be an analysis of frequency of service use prior to the attachment of costs to services. High cost services will be identified and their effect on skewedness of cost data analysed.
- The final dataset will be checked carefully before being passed (in an appropriate format) to the Senior Trial Economist for the purpose of calculation of ICERs and CEACs.
- A point estimate of the ICER, based on total costs and benefits will be calculated and results will be discussed between Senior Trial Economist and the Trial Economist prior to circulation to NWORTH and the wider trial team.
- The cost-effectiveness plane and CEAC curve will be analysed. Where the analysis involves QALYs, the cost per QALY result will be compared to the NICE threshold of £20,000 to £30,000. Where a cost-effectiveness ratio is expressed in terms of a point change on a disease specific instrument, results will be related to a clinically significant change, or case / non-case threshold.
- Modelling will be undertaken when costs and outcomes need to be extrapolated beyond the end date of the trial.

### *6.4.3 Report and publish*

Publication of results will be according to standard guidelines (e.g. Drummond, 1996; Ramsey *et al*., 2005; NICE, 2007). In general:

- The Senior Trial Economist/Guarantor will write the economic evaluation contribution to any trial protocol paper.
- Results and analysis will be written in a manner which is clear and relevant to policy makers and incorporated into the trial final report.
- Economic results will be published alongside clinical results where possible (e.g. BMJ pair of companion papers format).
- Sensible decisions will be made about how many significant figures should be reported in papers.

### *6.4.4 Critical review*

After each trial, CEPhI will evaluate, in consultation with NWORTH staff, strengths and weaknesses of the approach taken to economic analysis alongside that trial, and lessons for future trials. Lessons learned will be included in future training and development and incorporated into SOP reviews where appropriate.

# **Figure 1: Flow diagram for health economic evaluation in RCTs**

**Economic/Statistical analysis – undertake analyses (6.4.2)**

**Contribute to Trial Development Group (6.2.3)**

**TRIAL FUNDING AGREED**

**Review health economics literature (6.2.2)**

**Prepare an Economic Evaluation Analysis Plan (6.3.2)**

**Incorporate economic evaluation into paperless trial when necessary (6.3.3)**

**CEPhI Sustainability (6.1.1)**

**Role of CEPhI in securing research funding for trials (6.1.2)**

**Undertake critical review (6.4.4)**

**Report and publish (6.4.3)**

**Select CEPhI staff to be involved in the economic evaluation alongside a specific RCT (6.2.1)**

**Monitor collection of health economics data (6.3.4)**

**Present progress and interim results (6.3.7)**

**Economic/Statistical analysis – incorporate general principles (6.4.1)**

**Overview (6.1)**

**CEPhI activities for economic evaluation alongside a RCT prior to funding approval (6.2)**

**Funding agreed**

**CEPhI economic evaluation activities alongside a RCT during the data collection period (6.3)**

**CEPhI activities for economic evaluation alongside a RCT after the data collection period (6.4)**

**TIME PERIOD**

**CEPhI ACTIVITIES**

**Design and manage economic database (6.3.5)**

**Conduct a systematic review of the literature if not conducted under (6.2.2); otherwise update systematic review (6.3.6)**

**Select Health Economist(s) to join Trial Management Group (6.3.1)**

# Section 7: Staff Training and Development

The Senior Trial Economist will ensure that the research officer/s appointed to trials are trained and supported in the use of this and supporting SOPs. Trial coordinators and Data Managers will also be trained in the use of this and supporting SOPs.

Initial training will be in principle: the Director/Deputy Director of CEPhI will train Trial Health Economists, Trial Coordinators, and Data Managers in the use of this SOP. Staff will sign the training record.

Follow up training is given via practical application, staff will sign the training record once they have completed economic evaluation alongside a RCT.

# Section 8: References

Bang H and Tsiatis A A. 2000. Estimating medical costs with censored data. *Biometrika*, 87: 329-43.

Donaldson C, Mugford M and Vale L (Eds). 2002. Evidence based Health Economics: from effectiveness to efficiency in systematic review. London: BMJ Books.

Drummond M F. 1996. Guidelines for authors and peer reviewers of economic submissions to the BMJ. *British Medical Journal*, 313: 275-283. Available from URL: <http://bmj.bmjjornals.com/cgi/content/full/313/7052/275> (Accessed 18/05/2008).

Drummond M F and McGuire A (Eds). 2001. Economic evaluation in health care: merging theory with practice. Oxford: Oxford University Press.

Drummond M, Sculpher M J, Torrance G W, O’Brien B J, Stoddart G L. 2005. *Methods for the Economic Evaluation of Health Care Programmes.* Third Edition. Oxford: Oxford University Press.

Eggar M, Davey Smith G and Altman D (Eds). 2003. Systematic reviews in health care: meta-analysis in context. London: BMJ Publishing Group.

Glick H A, Doshi J A, Sonnad S S and Polsky D. 2007. Economic Evaluation in Clinical Trials. Oxford: Oxford University Press.

Groot Koerkamp B, Hunkink M, Stijnen T, Mannitt J K, Kuntz K M and Weinstein C. 2007. Limitations of acceptability curves for presenting uncertainty in cost-effectiveness analysis. *Medical Decision Making*, 27(2): 101-111.

Knapp M, Thorgrimsen L, Patel A, Spector A, Hallam A, Woods B, et al. 2006. Cognitive stimulation therapy for people with dementia: cost-effectiveness analysis. *British Journal of Psychiatry*, 188: 574-580.

National Institute for Health and Clinical Excellence (NICE). 2007. Review of the guide to the methods of technology appraisal (consultation document).

Office of Health Economics (OHE). 2008. Glossary - available from website at URL: <http://www.ohe.org/page/glossary.cfm> (Accessed 18/05/2008).

Ramsey S, Willke R, Briggs A, Brown R, Buxton M, Chawla A, Cook J, Glick H, Liljas B, Petitti D and Reed S. 2005. Good research practices for cost-effectiveness analysis alongside clinical trials: The ISPOR RCT-CEA task force report. *Value in Health*, 8(5): 521-533.

# **Section 9: Referenced SOPs**

**List of associated CEPhI/NWORTH SOPs (to be written)**

| **SOP No.** | **Title** | **Lead author** | **Due date** | **Status / (Priority)** |
| --- | --- | --- | --- | --- |
| EE.I | General overarching methodology | RTE | Dec 2007 | Draft (1) |
| EE.2 | Commissioning of economic evaluation into trial grant application | RTE/BH/DH | June 2009 | Planned (2) |
| EE.3 | Design of economic evaluation protocol document | BH/RTE/DH | March 2009 | Planned (1) |
| EE.4 | Incorporating economic evaluation into paperless trials | STY | June 2009 | Planned (2) |
| EE.5 | Collection of prescribed drugs information in trials | DH | March 2009 | Planned (1) |
| EE.6 | Systematic reviewing of economic evidence in trials | PL | June 2009 | Planned (2) |
| EE.7 | Data set cleaning | STY | March 2009 | Planned (1) |
| EE.8 | Health economics/statistical analysis in trials | DH/RTE/DR | March 2009 | Planned (1) |
| EE.9 | Preparing paired clinical and economic papers for publication | RTE/DH | June 2009 | Planned (2) |
| EE.10 | Modelling | DH/EF | June 2009 | Planned (2) |

# **Section** 10: Appendices

## Appendix 1: Training for this SOP

| **User** | **Trainer** | **Principle training** | **Practice training** | **Date Trained** | **Duration of training** | **Location of training** | **User signature** |
| --- | --- | --- | --- | --- | --- | --- | --- |
| Ian Russell |  |  |  |  |  |  |  |
| Rhiannon Whitaker |  |  |  |  |  |  |  |
| Karen Hughes |  |  |  |  |  |  |  |
| Daphne Russell |  |  |  |  |  |  |  |
| Seren Roberts |  |  |  |  |  |  |  |
| Emma Bedson |  |  |  |  |  |  |  |
| Angela Gliddon |  |  |  |  |  |  |  |
| Dyfrig Hughes |  |  |  |  |  |  |  |
| Bethan Henderson |  |  |  |  |  |  |  |
| Barry Hounsome |  |  |  |  |  |  |  |
| Rhiannon Tudor Edwards |  |  |  |  |  |  |  |
| David Ingledew |  |  |  |  |  |  |  |
| Michelle Williams |  |  |  |  |  |  |  |
| Pat Linck |  |  |  |  |  |  |  |
